# Food waste to biomass approach: A sustainable strategy for the production of alternative microbial culture media for filamentous fungi growth

**DOI:** 10.1371/journal.pone.0360680

**Published:** 2026-10-29

**Authors:** Hayet Cherrad, Soraya Djebara, Fatima Zohra El Kadi, Abdesselem Si Mohammed, Ahmed Megharbi, Rania Soumia Zellit, Mahmoud Helal, Rokayya Sami, Ola Abu Ali, Amal F. Alqurashi, Eman A. Abduljawad, Amera N. Alqahtani

**Affiliations:** 1 Department of Food Sciences, Faculty of Nature and Life Sciences, Abdelhamid Ben Badis University, Mostaganem, Algeria; 2 Department of Biological Sciences, Faculty of Natural Sciences and Life, University of Relizane, Relizane, Algeria; 3 Department of Biological Sciences, Faculty of Natural Sciences and Life, Djillali Liabes University, Sidi Bel Abbés, Algeria; 4 Department of Ecology and Environment, Faculty of Natural Sciences and Life, University of Relizane, Relizane, Algeria; 5 Department of Mechanical Engineering, Faculty of Engineering, Taif University, Taif, Saudi Arabia; 6 Department of Food Science and Nutrition, College of Sciences, Taif University, Taif, Saudi Arabia; 7 Department of Chemistry, College of Science, Taif University, Taif, Saudi Arabia; 8 Department of Clinical Nutrition, Faculty of Applied Medical Sciences, Umm Al-Qura University, Makkah, Saudi Arabia; 9 Department of Food and Nutrition, Faculty of Human Sciences and Design, King Abdulaziz University, Jeddah, Saudi Arabia; 10 Department of Biological Sciences, College of Science, University of Jeddah, Jeddah, Saudi Arabia; Amity University Noida, INDIA

## Abstract

The valorization of organic waste is still one of the challenges in the path towards environmental sustainability and a circular bioeconomy. This research aimed at assessing the ability of three substrates of organic residues (olive pomace, potato peels and the green seaweed *Ulva lactuca*) to be used as alternative culture media for filamentous fungi. Mycelial growth on these media was compared with the conventional Potato Dextrose Agar (PDA) and Sabouraud media using three fungal species namely *Fusarium oxysporum* (F1), *Fusarium solani* (F2) and *Alternaria alternata* (N2) as biological models. The alternative media were made from waste materials, supplemented or not with glucose, and sterilized prior to the use. The measurements of mycelial growth were taken every day for 8 days and the data analyzed by multi-factorial ANOVA. Mycelial growth in all the waste-based media was found to be comparable to commercial media. Highest overall growth was seen in commercial PDA and Sabouraud media (19.9% and 19.3%) respectively. The glucose enriched formulations showed 13.2% (olive pomace) and 11.0% (potato peel) rich media with the remaining media showing less. The percentages of olive pomace, potato peels and *U. lactuca* were 12.9%, 9.3% and 1.2%, respectively, without the addition of glucose. Growth was significantly higher with glucose supplementation, showing the beneficial effect of the exogenous C supply and intrinsic nutritional value of the substrates. ANOVA revealed that incubation time and medium type significantly influenced mycelial growth across all three strains (p < 2.2 × 10^−16^), with significant interactions between medium and time (F1: F = 3.91, p = 6.08 × 10^−10^, F2: F = 163.31, p < 2 × 10^−16^, N2: F = 44.30, p < 2 × 10^−16^). The overall analysis of the three strains also indicated highly significant main effect for strain (F = 645.22) and incubation time (F = 391.95) as well as highly significant main effect for culture medium (F = 44.18) (all p < 2.2 × 10^−16^). Variations in texture and color that can be seen in the different media, however, may influence the macroscopic appearance of the mycelium and identification. Hence, these alternative media are recommended for post identification, subculture and preservation of fungal strains especially.

## Introduction

Organic waste management is one of the more critical environmental issues, related to urbanization, increased agricultural and industrial activity and population growth. Every year, billions of metric tons of organic waste are produced globally, with most still being burned or buried, causing high greenhouse gas emissions, loss of renewable raw materials, and soiling and polluting soil and water resources [1,2]. In response to this increased demand to limit these environmental impacts, alternative solutions and strategies have emerged that specifically incorporate both the bioeconomy and circular economy. In these schemes, the waste is no longer viewed as an end product but as a bioresource in its own right [3,4]. In fact, the recent advances of microbial fermentation, industrial biotechnology and the ever-growing realm of biorefineries have helped to improve the conversion potential of organic waste into renewable products, offering new opportunities in the sustainable management of not only agricultural and agri-food wastes but also the urban organic fractions [5]. Biodegradable waste is particularly attractive for this valorization pathway because it provides a dual benefit: it is both quantitatively abundant and qualitatively rich, comprising macromolecules, fibers, lipids, minerals, and a host of bioactive compounds. This potential is driving strong interest for compounds and applications across a wide range of industrial sectors such as agri-food, biotechnology, pharmaceuticals and specialty chemicals, applied in the production of enzymes, organic acids, single cell proteins, and bioplastics [6]. On a larger scale, this method lessens the expense typically linked with waste disposal and also cuts fossil-based feedstock dependency and greenhouse gas emissions of traditional production methods [4].

Amongst these organic resources is algal biomass. Excessive growth of macro and micro algae, mainly caused by eutrophication due to high concentrations of excess nitrogen and phosphorus from agricultural, industrial and urban sources which consequently cause imbalance in aquatic ecosystems. [7,8]. Poor water quality, loss of dissolved oxygen and decline in aquatic habitat, as well as substantial economic losses in the fisheries, aquaculture and tourism industries, and higher costs of aquatic management and rehabilitation result [9,10]. The polysaccharides, pigments and other bioactive compounds contained in this biomass are very rich and are highly recyclable, especially for biomaterials, nutraceuticals, biofuels, biofertilizers [11,12] and water remediation [13,14]. In this context, filamentous fungi have been found to be especially promising microorganisms in the therapy of organic wastes [15–17]. These microorganisms produce large amounts of extracellular hydrolases such as pectinases, cellulases, amylases, hemicellulases, and ligninases [18]. The concurrent activity of these enzymes enables them to perform very well at breaking down the plant biomass and agri-food residues that are known as recalcitrant polymeric networks [19]. The collective action of these enzymes renders them particularly adept at dismantling the recalcitrant polymeric networks found in plant biomass and agri‑food residues. This inherent metabolic diversity has paved the way for their expanded utilization in a range of biotechnological contexts, from the production of fungal biomass and industrial enzymes to the synthesis of organic acids and natural pigments [20]. Filamentous fungi have now earned their stripes as pivotal biological players in the circular bioeconomy not least because they combine high conversion yields with low input costs and an impressive tolerance for heterogeneous waste streams [21]. This strategy aims not only to limit the pollution associated with waste accumulation, but also to enhance the sustainability of biotechnological processes by making use of under-utilized local resources.

## Materials and methods

As part of this work, three types of waste from different sources were selected due to their abundance, low cost and potential for recovery as alternative substrates for the cultivation of filamentous fungi, and because they are widely available in Algeria, a major producer of olive oil and potatoes, with an extensive coastline where macroalgae proliferate naturally.

### Olive pomace

It is a by-product of olive oil extraction. It consists of fragments of pulp, skins and crushed pits, forming a lignocellulosic residue rich in fiber, residual sugars and phenolic compounds [22,23]. It is very abundant the Mediterranean region and is a very strategic substrate that should be used, especially in the field of biotechnological applications in the circular economy.

### Preparation of olive pomace flour

In the olive oil production area of Mascara (western Algeria), olive pomace is systematically gathered during the olive oil season (December – February 2024), thus reducing the amount of olive pomace in the environment and the risk of contamination to local ecosystems. The crude residues were then initially dried in the oven at 45 °C. This is particularly crucial to ensure the stability of the substrate and to avoid microbial growth that may change the substrate's composition. The pomace was mechanically ground after drying in order to obtain a fine and even powder which was later screened to eliminate the debris and ensure uniformity in particle size. The flour obtained from this (Fig 1) was kept in an airtight container in a dry dark place until it was used. It had higher solubility in distilled water, an important characteristic when it is to be used as the medium substrate for the culture.

**Fig 1:**
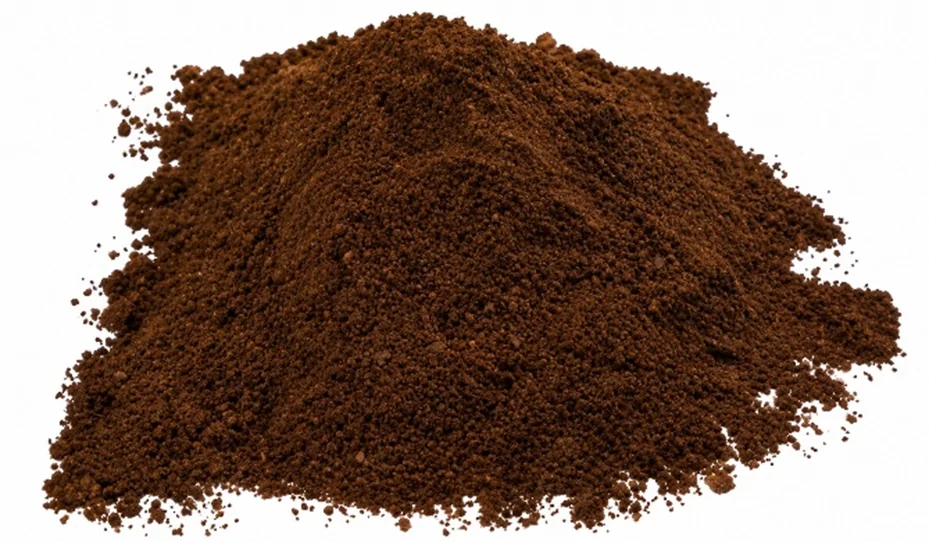
Olive pomace flour.

### Preparation of the culture medium based on olive pomace

Two distinct processes were employed in the formulation of the culture media, initially two suspensions were made by dissolving 20 g and 10 g of powdered olive pomace in 250 mL of distilled water, respectively. As an extra carbon source, 5 g and 2.5 g of glucose were added. In order to assess olive pomace’s capacity to function as the special carbon substrate for fungal growth, control media were simultaneously made using the same procedure but without the addition of glucose, Following total dissolution, the suspensions pH was adjusted to 5.6, which is known to be ideal for the growth of many filamentous fungi. In order to guarantee gelation and produce solid medium appropriate for the experimental fungal cultures, 5 g of agar was finally added to each batch. Following autoclaving, all preparations were kept at 4°C until use.

### Green seaweed (*Ulva lactuca*)

*U. lactuca*, a common green macro alga on coastal shorelines. Its proliferation, often is frequently accelerated by eutrophication, leads to massive accumulations on beaches which have negative effects on the ecosystem and human health [24,25]. This biomass is desirable resource to divert from its polluting role toward sustainable biotechnological applications since it is readily harvestable and rich in proteins, minerals, and polysaccharides

### Preparation of algae flour

*U. lactuca* was collected at the end of March 2024 at the coast of Oran, western Algeria, and was immediately washed with a few changes of distilled water to remove any debris, sediment, and epiphytes that might affect the quality of the seaweed. After being cleaned, it was allowed to dry outdoors for three weeks, away from light and moisture. After that, the dried biomass was milled in into a uniformly fine powder. In order to eliminate coarse particles and guarantee a uniform particle size, a crucial requirement for its use as an experimental substrate, this algal flour was sieved. The final product was the powder (Fig 2) which was packaged and stored in light and moisture-resistant containers.

**Fig 2:**
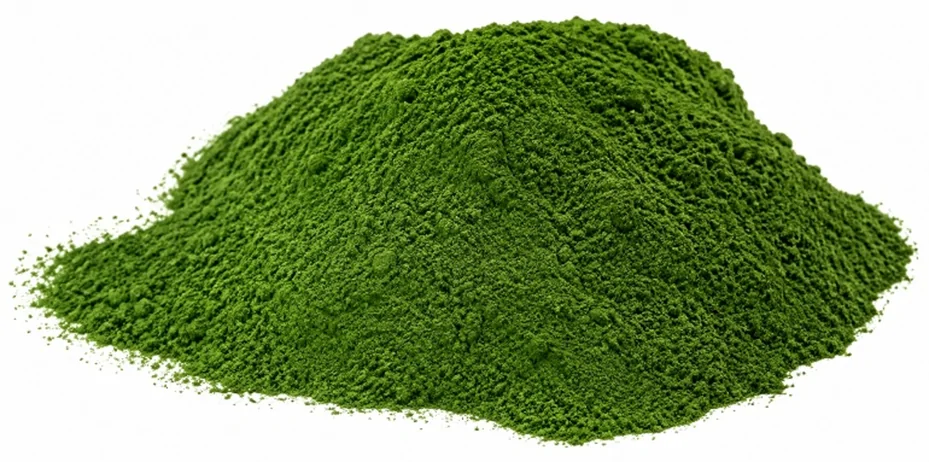
*Ulva lactuca* algae flour.

### Preparation of the culture medium based on algae

10 g of *U. lactuca* powder were dissolved in 250 mL of distilled water to create two different formations. 10 g of glucose were added to the initial solution to enrich it and provide an extra carbon source that would promote fungal growth. The purpose of the second, which did not include glucose, was to assess algal biomass’s capacity to serve at the exclusive carbon substrate. Following the uniform dissolution of the powders, each solutions pH was adjusted to 5.6, which is ideal for the growth of numerous filamentous fungi. In the order to facilitate gelation and produce solid media appropriate for experimental culturing, 5 g of agar was added to each mixture. Before being employed in the experiment, all preparations were autoclaved and kept at 4°C.

### Potato peels

Potato peels (Fig 3) are a major source of food waste that comes from both residential and industrial processing. They are a great nutrient substrate that may be utilized in a variety of microbial activities since they are mostly made of starch, dietary fiber, and micronutrients [26]. Their valorization reduces food waste and provides a cost-effective substitute for traditional culture media.

**Fig 3:**
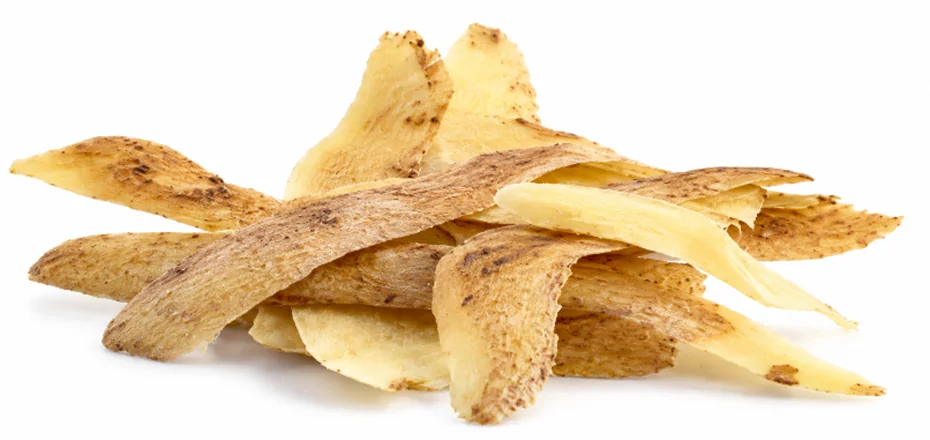
Potato peels.

### Preparation of the culture medium based on potato peels

The potato peels were carefully washed with distilled water to ensure the removal of dirt or impurities from them and were then collected from the residential waste. Cleaned potato peels were used without drying or grinding them for the preparation of culture medium. The cleaned potato peels were not dried or ground, and were thus used in the preparation of the culture medium. 200g of peels was boiled in 1,000mL of distilled water to remove nutrients and soluble substances. After boiling, the obtained broth was filtered out to provide a clear, uniform extract. Two 250 mL aliquots of this extract were separated. 5 g of glucose were added to the initial aliquot as an extra carbon source, and then 5 g of agar were added to let the medium solidify. In order to determine whether potato peels could provide a sufficient carbon supply, the second aliquot was prepared containing 5 g of agar and absolutely no glucose. The pH of each preparation was adjusted to 5.6, considered optimal for the development of filamentous fungi. Finally, all preparations were sterilized by autoclaving and stored at 4°C until use.

### Fungal strains tested

As part of this study, three species of microscopic filamentous fungi were selected (Fig 4): *Fusarium oxysporum* (F1) (Genbank ascension number PX998659), *Fusarium solani* (F2) (Genbank ascension number PX998649) and *Alternaria alternata* (N2) (Genbank ascension number MN542657). The fungal strains were originally isolated from rhizosphere soil collected from a tomato field. These species were chosen because of their agronomic and phyto-pathological importance, as they are the agents responsible for major fungal diseases affecting a wide range of economically important crops [27,28]. Their ability to grow on various organic substrates also makes them relevant models for evaluating the potential for recycling agri-food and environmental waste as alternative culture media. The colonies of the studied strains were purified through successive sub-culturing steps, followed by single-spore isolation to obtain genetically homogeneous cultures [29]. All the three strains were maintained on Czapek Dox’s agar slants at 4°C and regularly transferred to fresh medium to ensure viability and morphological stability until use [30].

**Fig 4:**
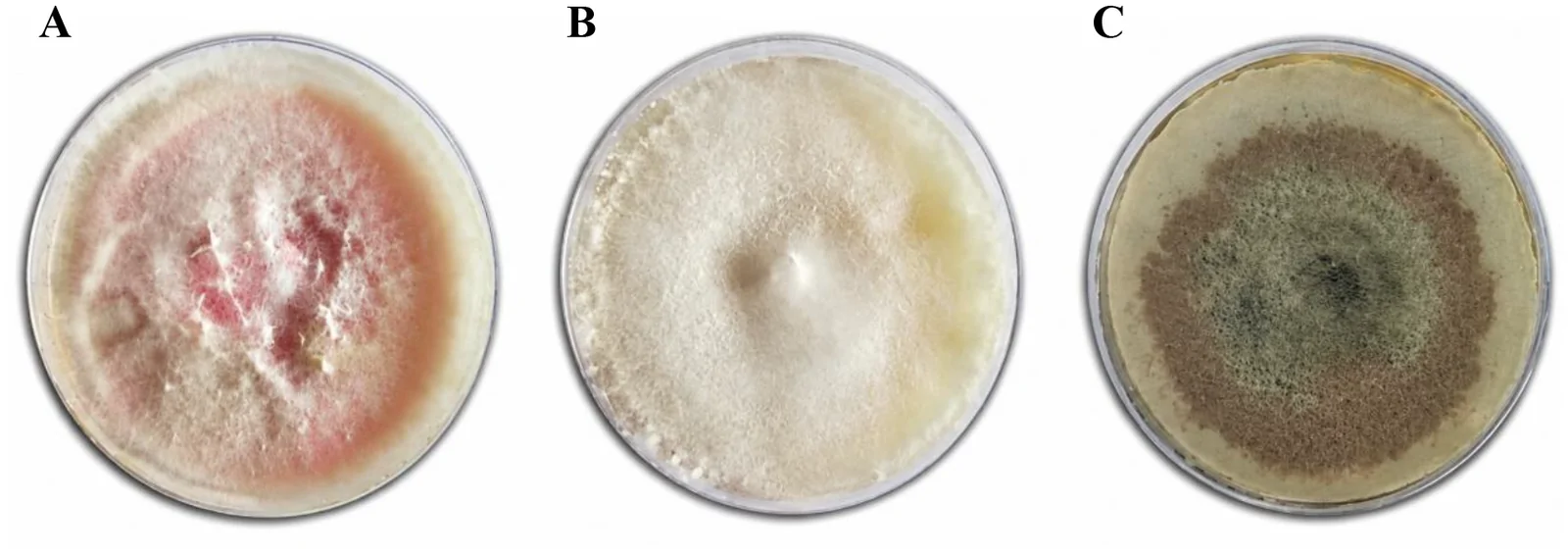
Fungal strains tested. A – F. oxysporum (F1); B – F. solani (F2); C – A. alternata (N2).

### Results reading

The cultures were incubated at 28 ± 1 °C for 8 days in a static incubator with controlled humidity, under sterile conditions and in the dark [30,31]. Three independent replicates were used for each test. Mycelial growth was monitored by measuring two perpendicular diameters (D1 and D2) for each colony in centimeters. Readings were taken at regular intervals, expressed in days, and continued until the colonies reached the edges of the Petri dish. The values obtained on the different experimental media were then systematically compared with those of the control in order to evaluate the effect of the substrates tested on fungal growth.

### Statistical analysis

The most effective and modern way to graphically represent results is using a box-and-whisker plot. In addition, the means of different groups were plotted using a mean plot.

The results were expressed as mean ± Standard Deviation (SD). A multi-way analysis of variance (ANOVA) test was applied for between two categorical factors, and the independent variable (diameters, days and medium), considering p < 0.05 as statistically signifcant. Statistical analysis was performed on the data using R software 4.0.0.

## Results

### Culture media

The culture media were prepared following a simplified protocol. Distilled water was supplemented with fine powdered substrates derived from potato peel infusion, *U. lactuca*, or olive pomace. A defined quantity of agar was subsequently incorporated, with or without the addition of glucose. Each medium showed a uniform mixture, with their varying opacities and complete dissolution of organic particulates dependent on the type of waste used. After sterilization all three formulations of the media gelated within 20–30 minutes indicating successful gelation. Importantly, the media could also be re-liquefied by heating for 30 minutes in a water bath and it was used again and again, indicating thermal stability and their ability to be used repeatedly in routine laboratory uses. The gels were plated on Petri dishes and showed a surface for seeding and uniformity of texture similar to commercial media. This is of great interest since it reflects the practical usefulness and applicability of the developed preparation method in the context of the standard experimental protocol since the physical and functional characteristics of the obtained culture media are comparable to commercially available ones.

### Strain F1

The F1 strain showed significant growth in all the formulations tested. Specifically, this strain under the alkaline and acidic substrates based on olive pomace, algae and potato peel infusion showed an interesting capacity to absorb the available nutrients for the development of its mycelial. But growth was slightly superior in sugar supplemented conditions and increased mycelial density and colonization rate of colonies were observed. Conversely, the potato peel based medium without sugar promoted substantially poorer development (Fig 5).

**Fig 5:**
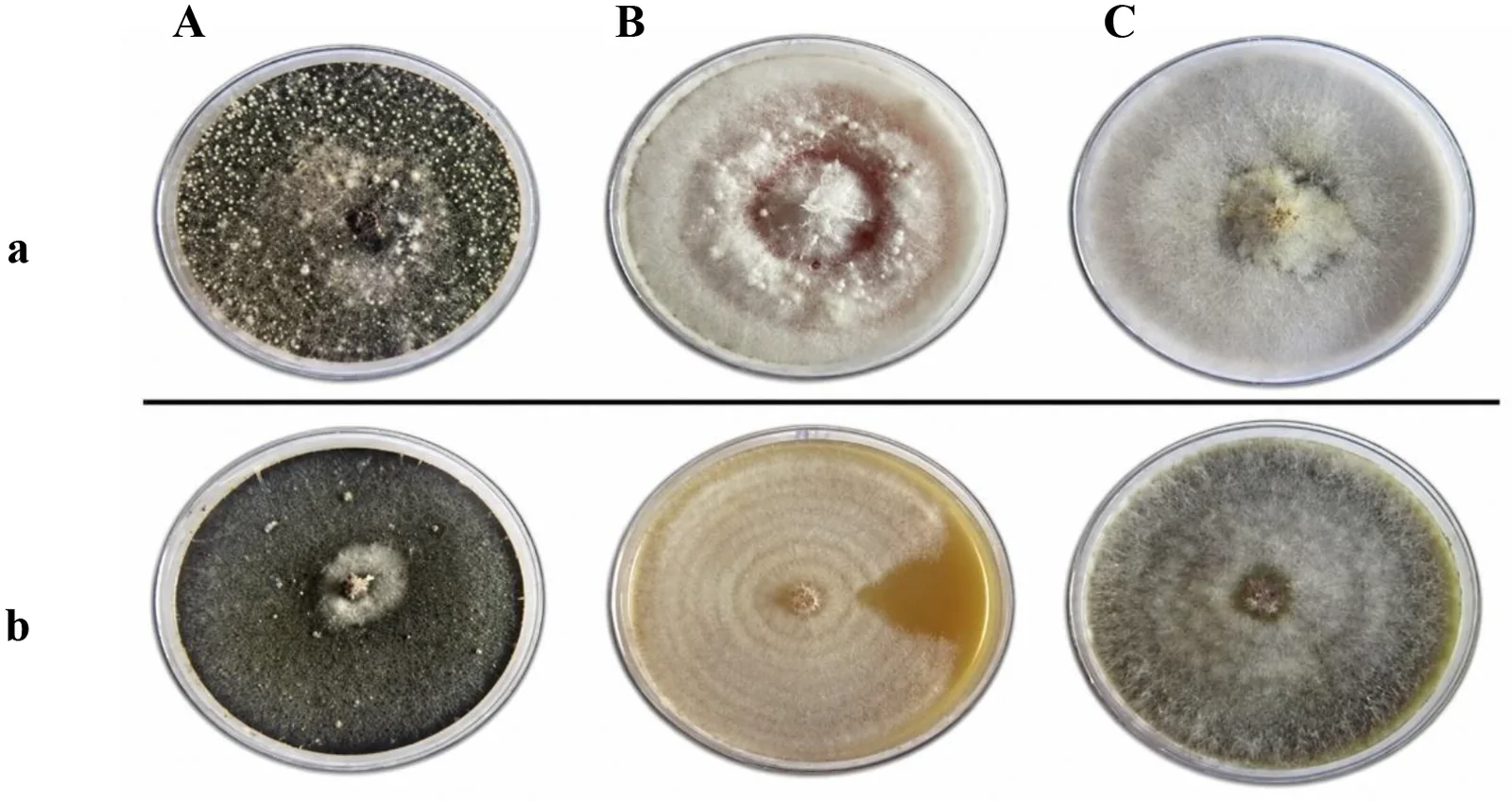
Growth of F1 strain on the three media. (A): Olive pomace medium; (B): Potato peels medium; (C): Algae medium, (a): Medium with glucose; (b): Medium without glucose.

This finding implies that, while olive pomace and *U. lactuca* seaweed is naturally high in organic compounds and vital nutrients, the addition of sugar offers readily absorbed energy source that encourages mycelial growth. Put differently., sugar functions as a metabolic supplement that improves the use of nutrients found in organic substrates, resulting in longer lasting mycelial activity. The F1 strains progressive use of nutrients in all tested conditions, whether or not they are enhanced with sugar, is shown by the box plots showing growth over eight days (Fig 6).

**Fig 6:**
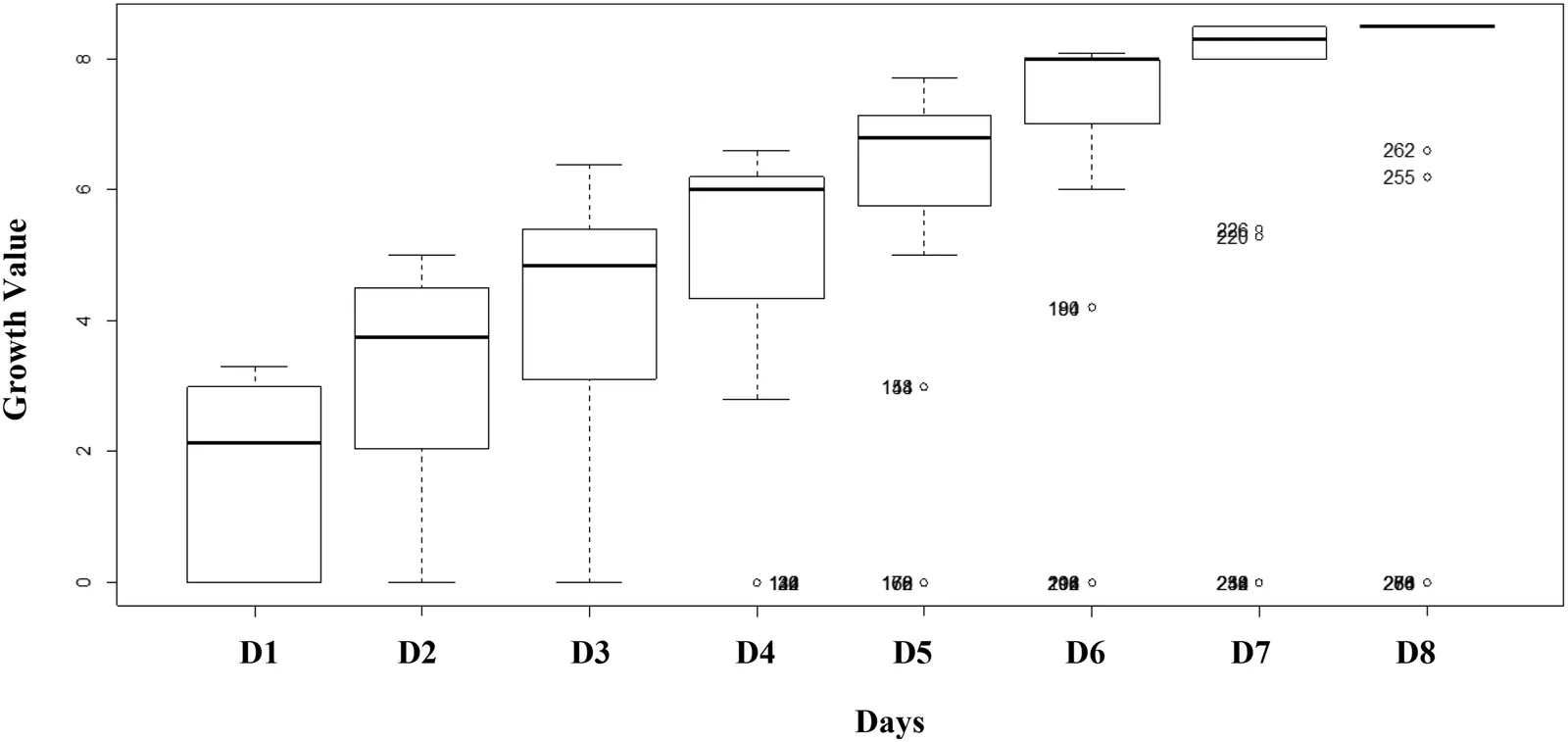
Temporal box plots for strain F1.

Fungal growth was generally more favorable on sugar enriched substrates then on non-enriched ones. The symmetry seen in the whisker boxes (Fig 7), which represents an uneven distribution of growth data related to the stimulating impact of sugar, makes this difference very clear.

**Fig 7:**
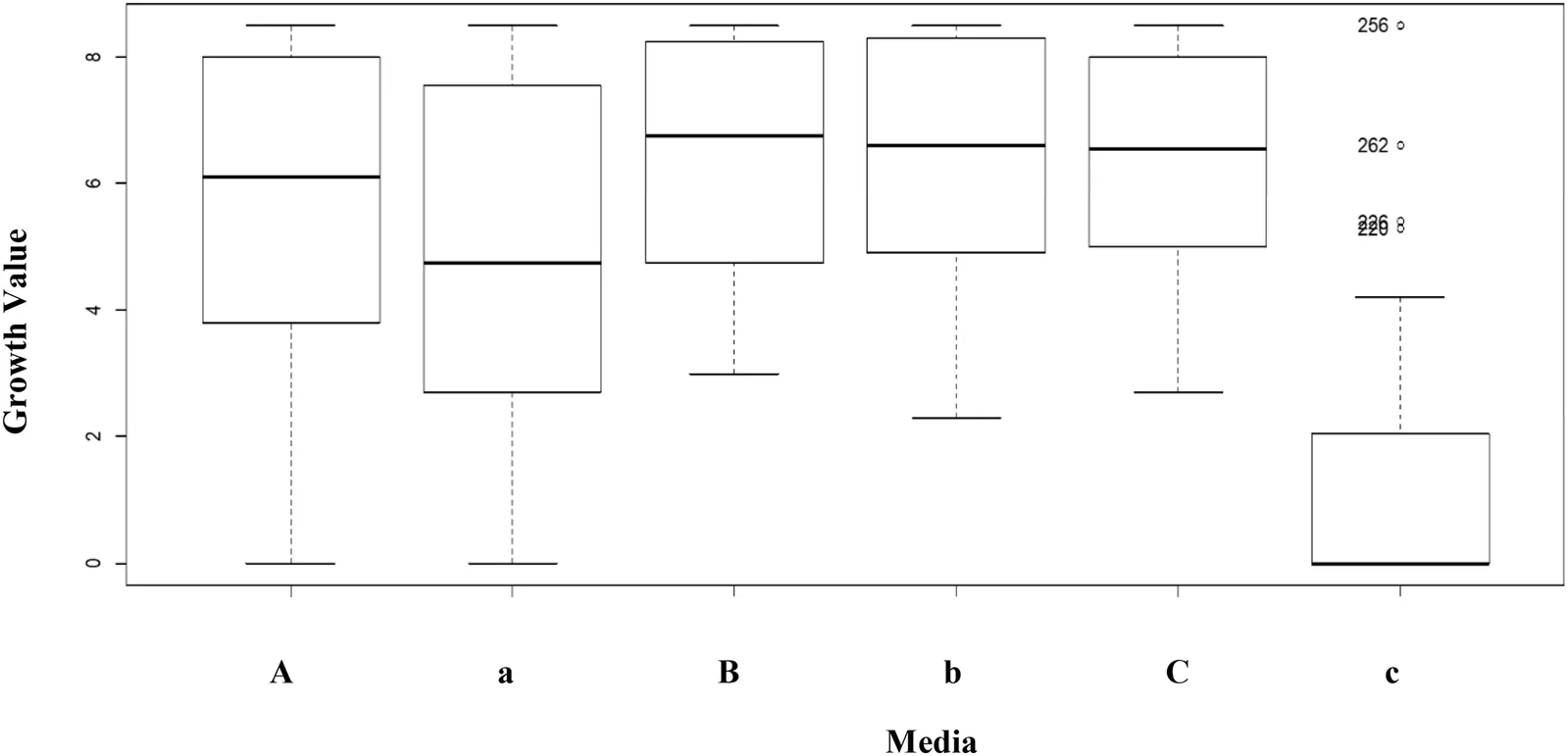
Box plots comparing different culture media for strain F1. (A): Algae with glucose; (a): Algae without glucose; (B): Olive pomace with glucose; (b): Olive pomace without glucose; (C): Potato peels with glucose; (c): Potato peels without glucose.

Further, the graph of averages (Fig 8) indicates and significant increase in the mycelial growth rate, which is in favor of the hypothesis that sugar is a readily-digested energy source for fungi.

**Fig 8:**
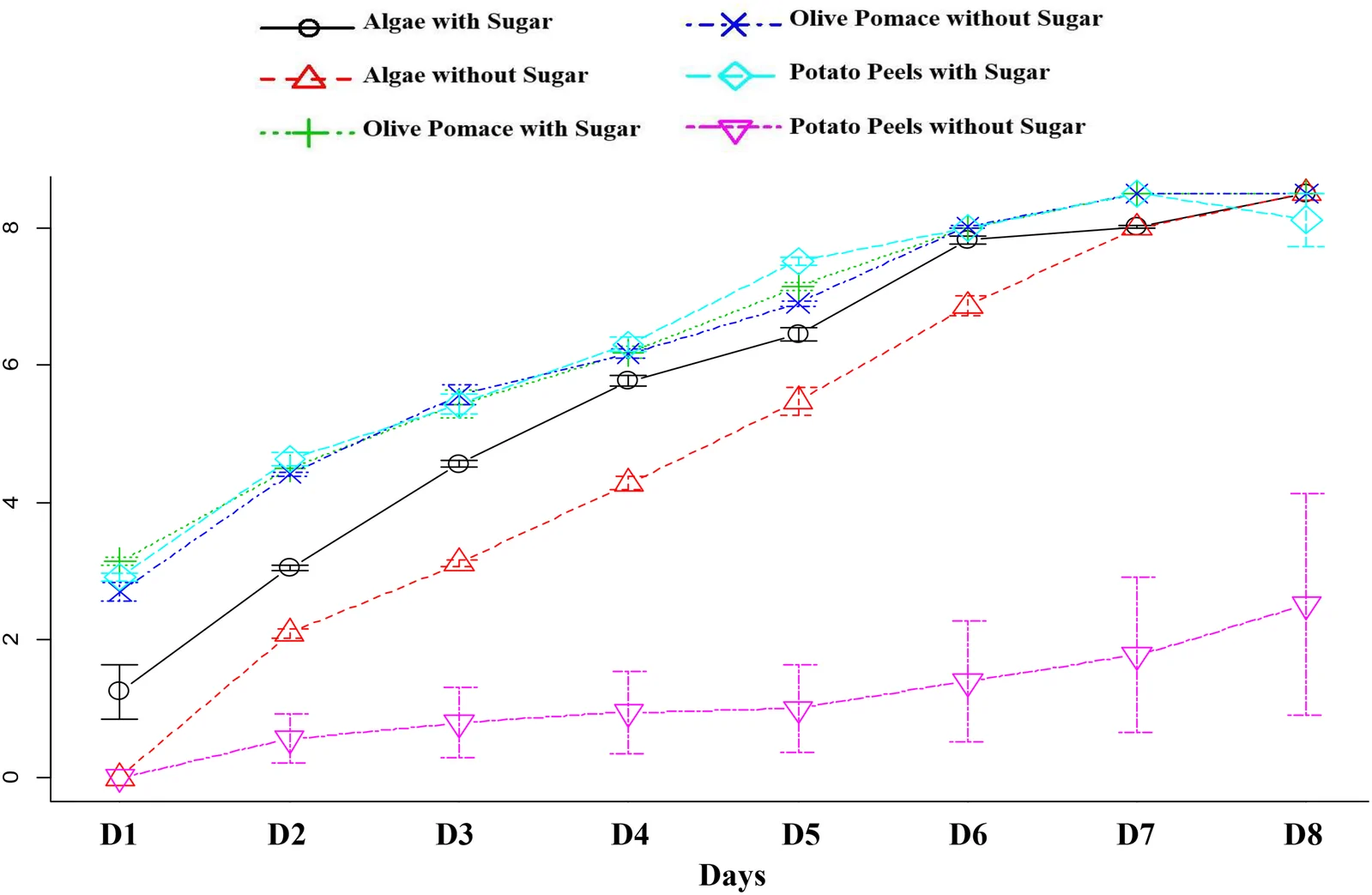
Mean diameters on the three-culture media (strain F1).

Multifactorial variance analysis to show that highly significant differences exist among the tested media (P < 2.2 < 10–16) and that the composition of the substrate is an important factor in growth. In addition, the time x medium interaction is highly significant (Table 1), indicating that the dynamics of mycelial development is very dependent on the interaction of these two experimental factors.

**Table 1:** Analysis of variance (strain F1).

Strain F1					
	SumSq	Df	F value	e Pr(>F)	
Diameter	0	1	0.0037	0.9515	
Days	1052.02	7	153.32	_<2.2_e-16	***
Medium	1021.14	5	208.3476	_<2.2_e-16	***
Diameter: Days	0.22	7	0.0314	1	
Diameter: Medium	0.3	5	0.0605	0.9976	
Days: Medium	134.22	35	3.9121	_6.08_e-10	***
Diameter: Days: Medium	1.74	35	0.0508	1	
Diameter	188.2	192			

Signif. codes: 0 ‘***’ 0.001 ‘**’ 0.01 ‘*’ 0.05 ‘.’ 0.1’ ‘1.

### Strain F2

The dietary needs of strain F2 were substantially greater than those of strain F1. mycelial density stayed comparatively low in sugar free media, indicating that this strain needs larger concentrations of readily absorbed carbohydrates for optimum growth. The results obtained with the media containing glucose as an enriching substrate, where a high mycelial density was observed, confirm the theory, except for the olive pomace based medium. However, even though the mycelium had spread throughout the entire surface of the petri dish (Fig 9), the thallus was flattened and slightly discolored suggesting a physiological reaction to the substrate. These findings demonstrate strain F2’s heightened reliance on carbohydrates inputs under the investigated experimental circumstances.

**Fig 9:**
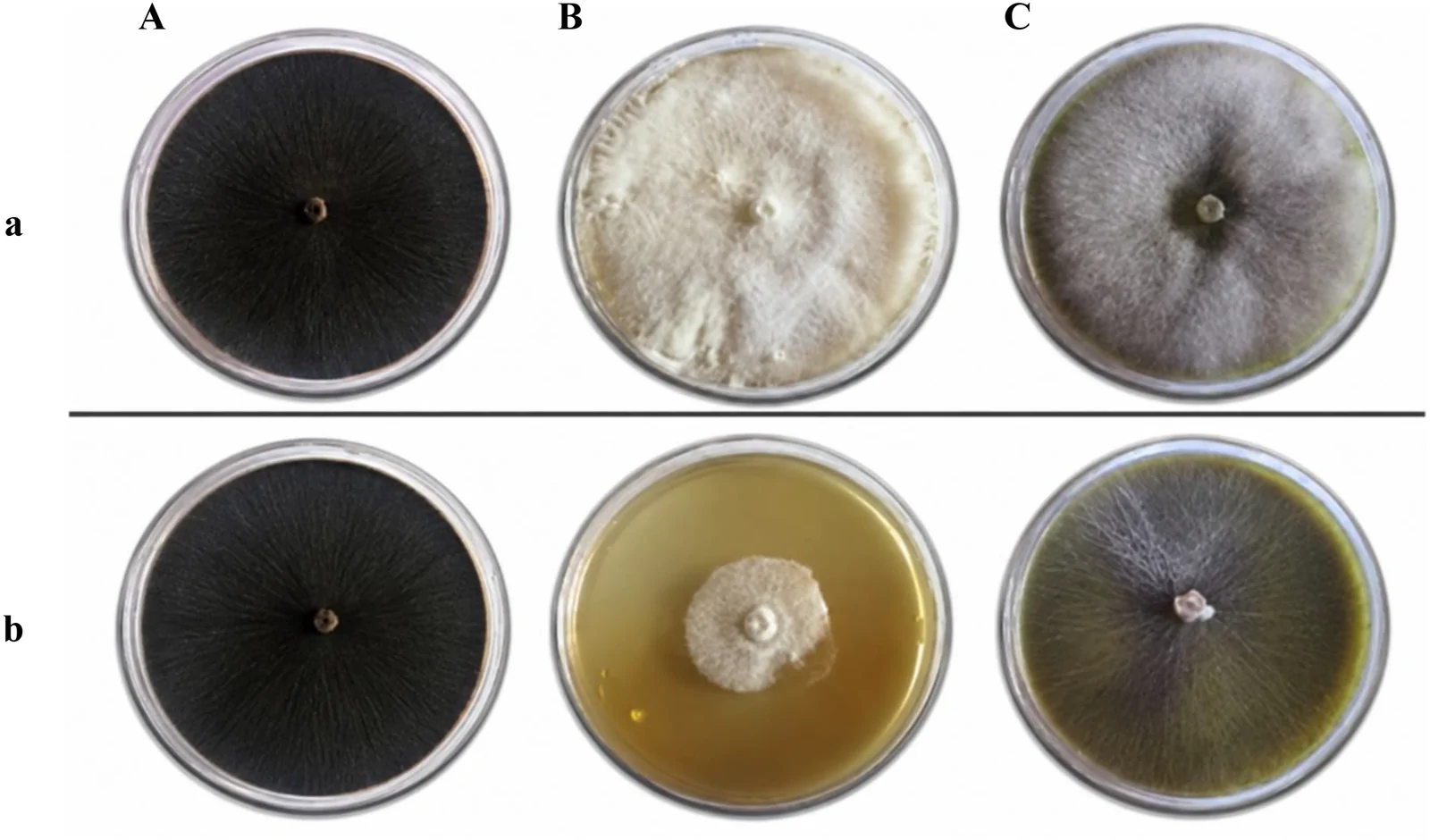
Growth of F2 strain on the three media. (A): Olive pomace medium; (B): Potato peels medium; (C): Algae medium (a): Medium with glucose; (b): Medium without glucose.

The ability of the strain F2 to progressively multiply on various media, whether or not they are loaded with sugar, during the course of the eight days of incubation is demonstrated by the regression of surface areas shown in the box plots (Fig 10). In other words, strain F2 shows the capacity to adapt and expand gradually overtime, despite the fact that growth may initially seem feeble or restricted.

**Fig 10:**
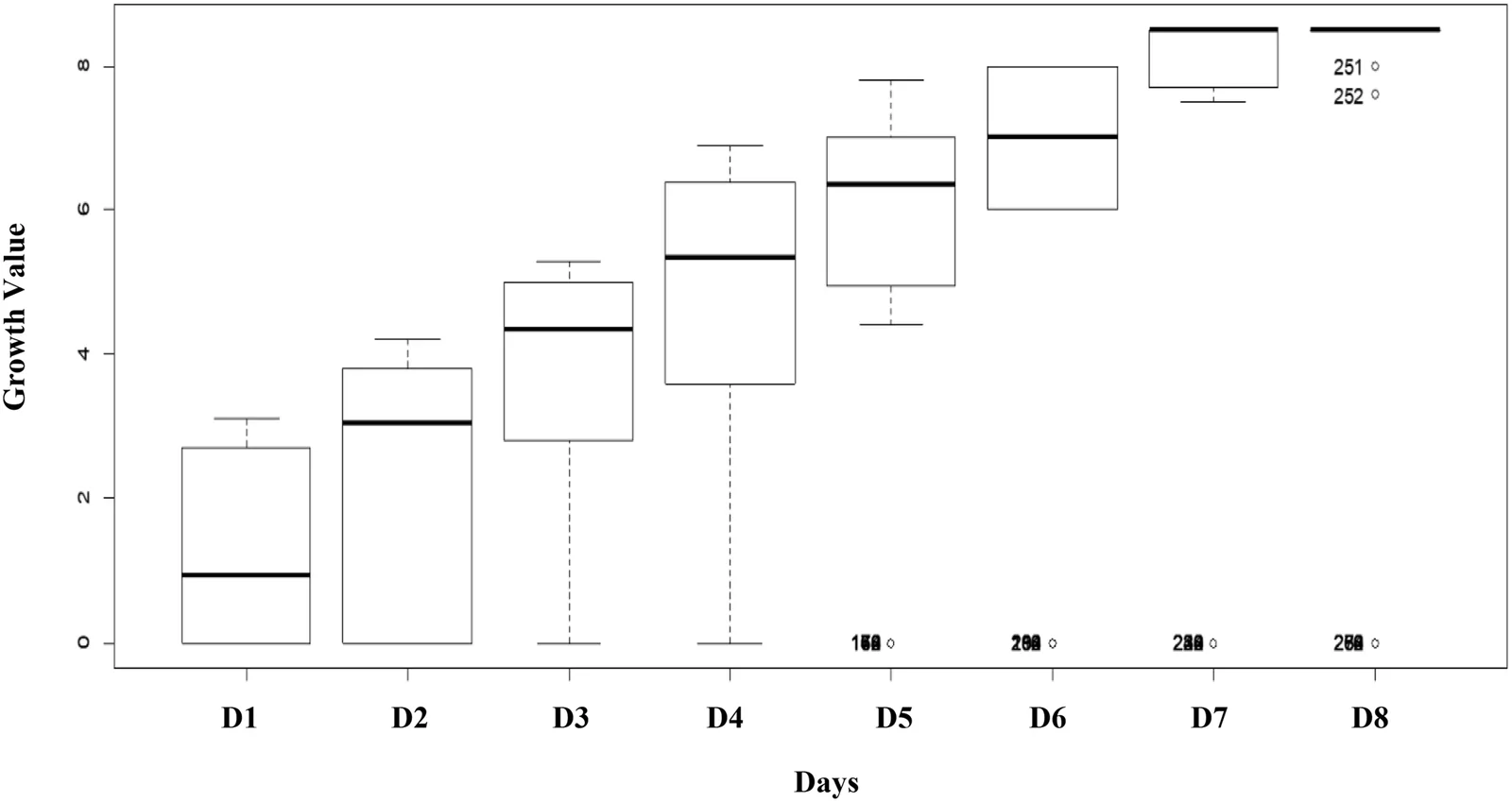
Temporal box plots for strain F2.

We may discover important trends related to the development dynamics on the studied media by visually analyzing this box plots, which show significant variability in mycelial growth between substrates (Fig 11).

**Fig 11:**
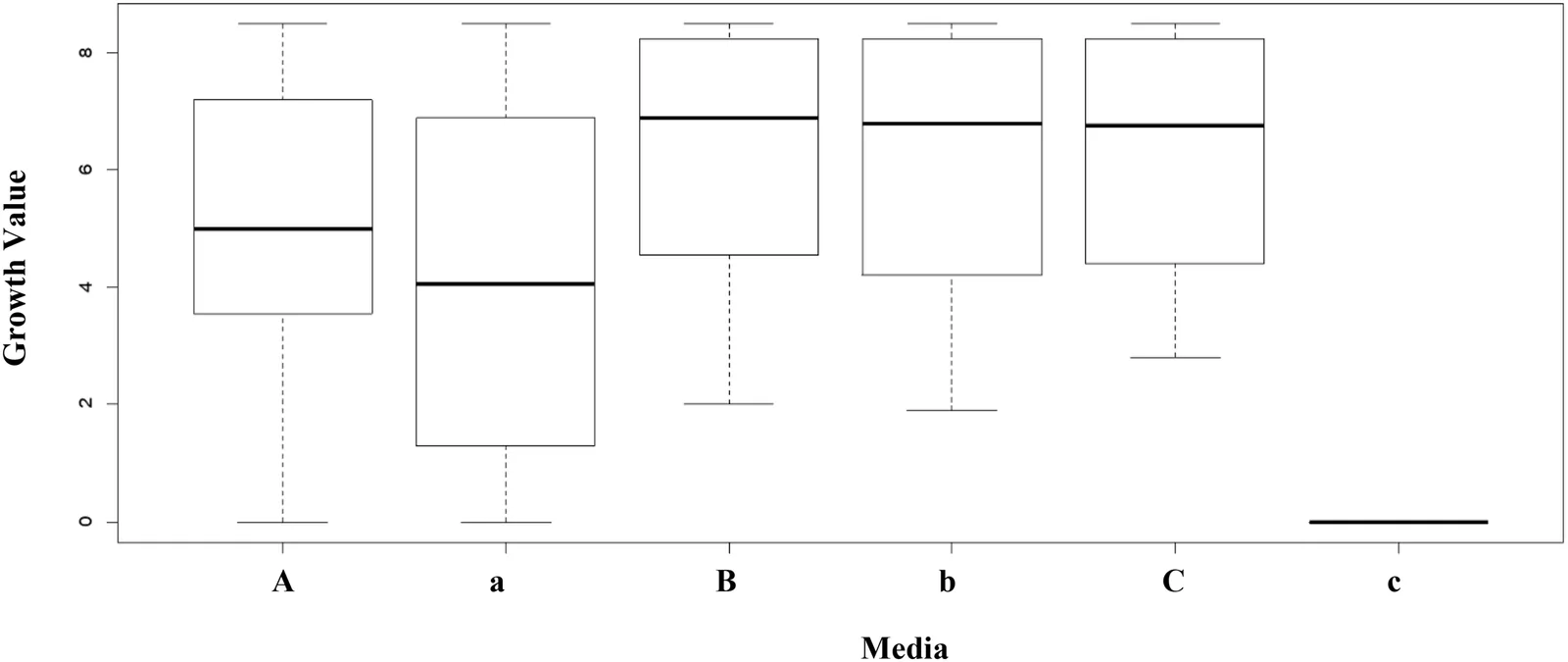
Box plots comparing different culture media for strain N2. (A): Algae with glucose; (a): Algae without glucose; (B): Olive pomace with glucose; (b): Olive pomace without glucose; (C): Potato peels with glucose; (c): Potato peels without glucose.

Supported by the graph of average (Fig 12) which makes it evident how the F2 strain depends on the addition of glucose for nourishment. growth was considerably delayed in the absence of glucose, especially on the medium based on potato peels where the mycelial diameter remained modest after eight days of incubation. these findings support the critical function that are similar carbohydrates in promoting strain F2 ideal growth.

**Fig 12:**
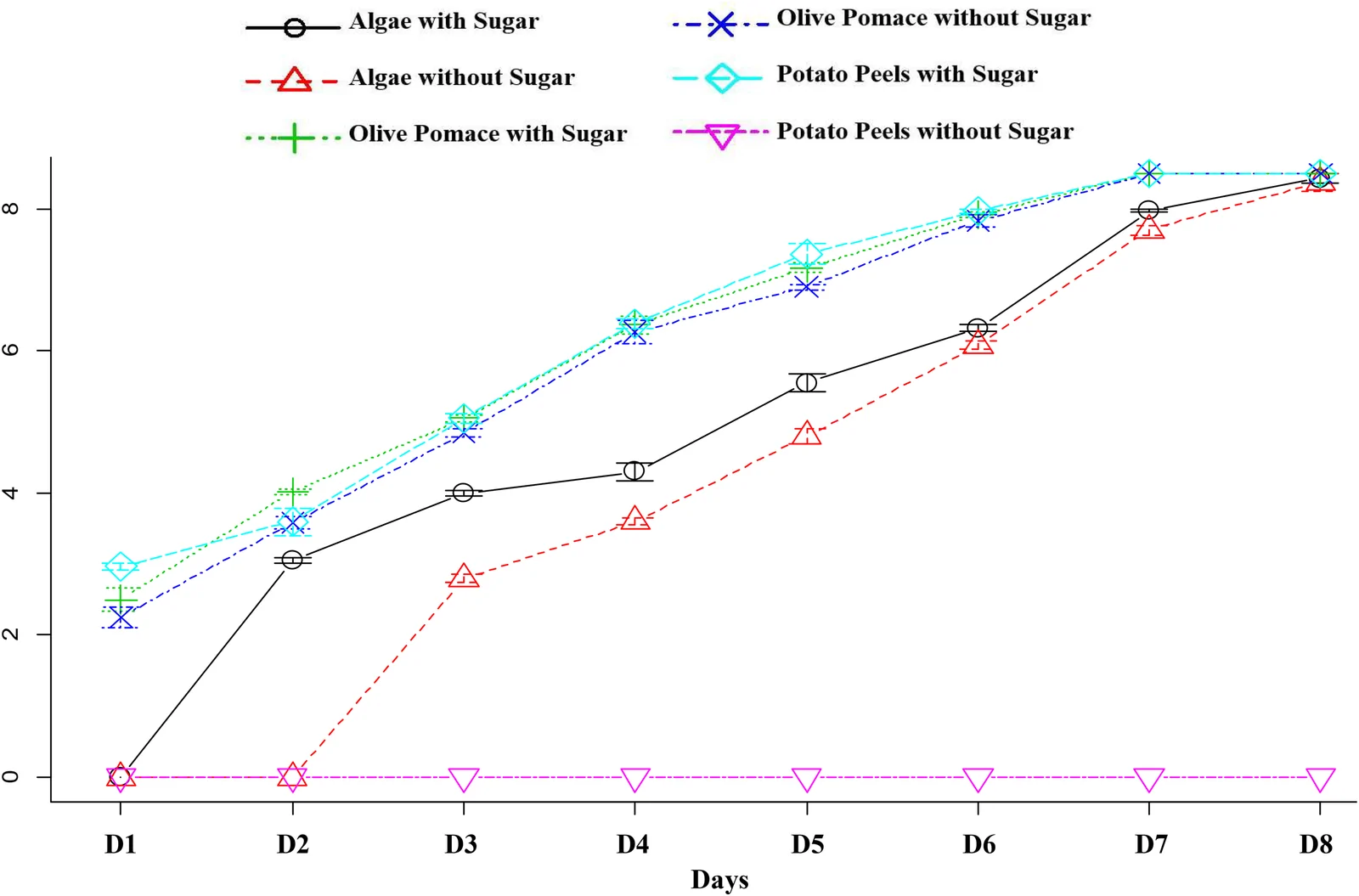
Mean diameters on the three-culture media (Strain F2).

Both the nutrient absorption time and the test media composition showed extremely significant differences (P < 2.2 × 10^-16^) according to multifactorial analysis of variance (ANOVA) these findings show notable differences in culture medium emphasizing the combined impact of time and substrate composition on mycelial development, kinetic (Table 2).

**Table 2:** Analysis of variance (Strain F2).

Strain F2					
	SumSq	Df	F value	e Pr(>F)	
Diameter	0.01	2	0.1188	0.8881	
Days	983.17	7	3334.887	_<2_e-^16^	***
Medium	1389.06	5	6596.276	_<2_e-^16^	***
Diameter: Days	0.04	8	0.1231	0.9983	
Diameter: Medium	0.18	9	0.4794	0.8873	
Days: Medium	240.73	35	163.307	_<2_e-^16^	***
Diameter: Days: Medium	0.28	32	0.2055	1	
Diameter	7.96	189			

Signif. codes: 0 ‘***’ 0.001 ‘**’ 0.01 ‘*’ 0.05 ‘.’ 0.1”1.

### Strain N2

Strain and showed a universal capacity to utilize various nutrient sources by growing in all examined medium. However the thallus seemed thin and underdeveloped in the glucose free potato peel based medium, which showed somewhat slower growth (Fig 13). These finding highlights strain N2 capacity to adapt to available substrate and implies that it is somewhat dependent on carbohydrate inputs to attain optimal mycelial growth.

**Fig 13:**
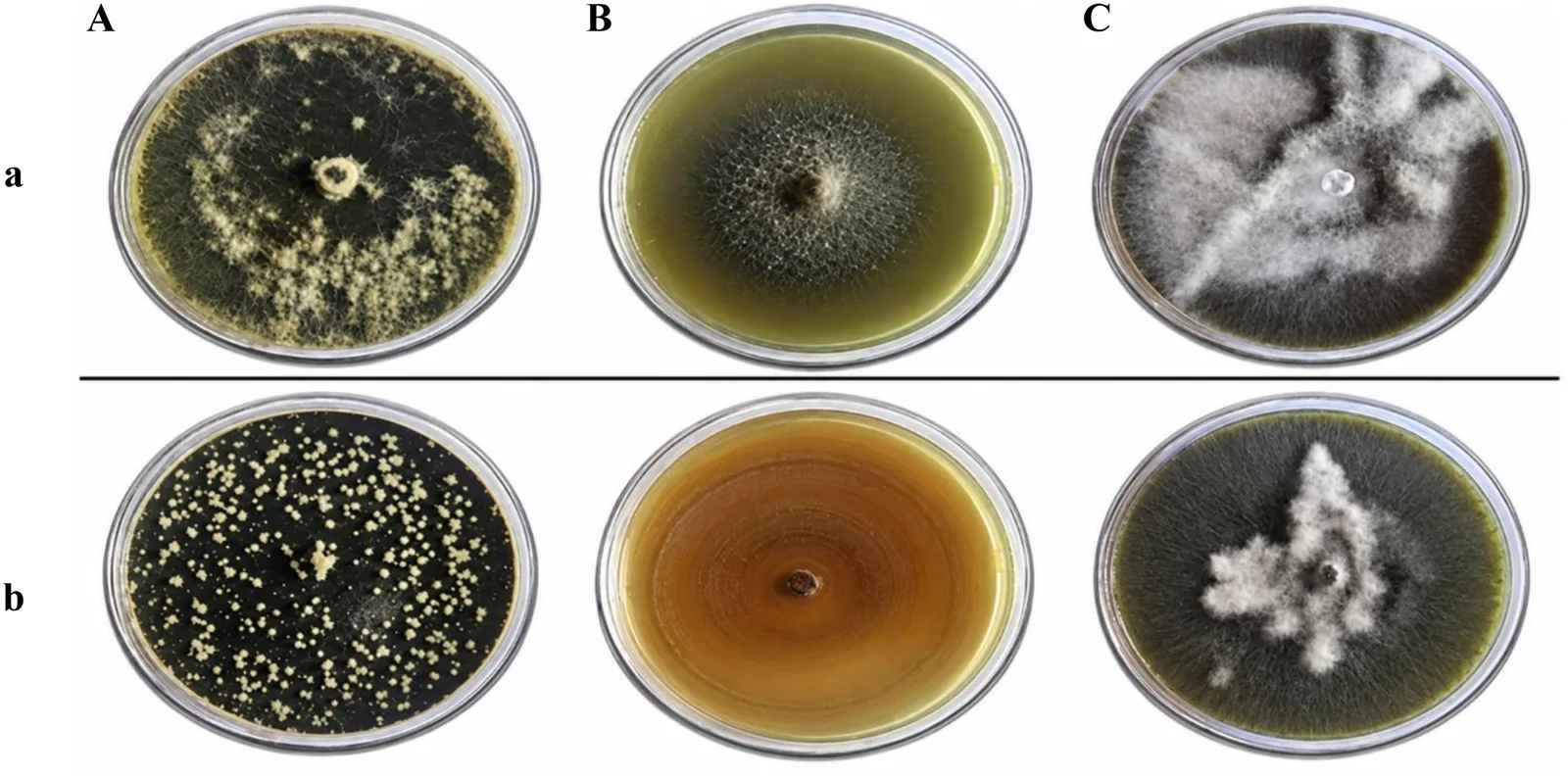
Growth of N2 strain on the three media. (A): Olive pomace medium; (B): Potato peels medium; (C): Algae medium(a): Medium with glucose; (b): Medium without glucose.

It is evident from an analysis of the box plot shown in (Fig 14) that strain N2 effectively uses the nutrients present in all tested media, whether or not they are sugar enriched for the course of the eight-day incubation period.

**Fig 14:**
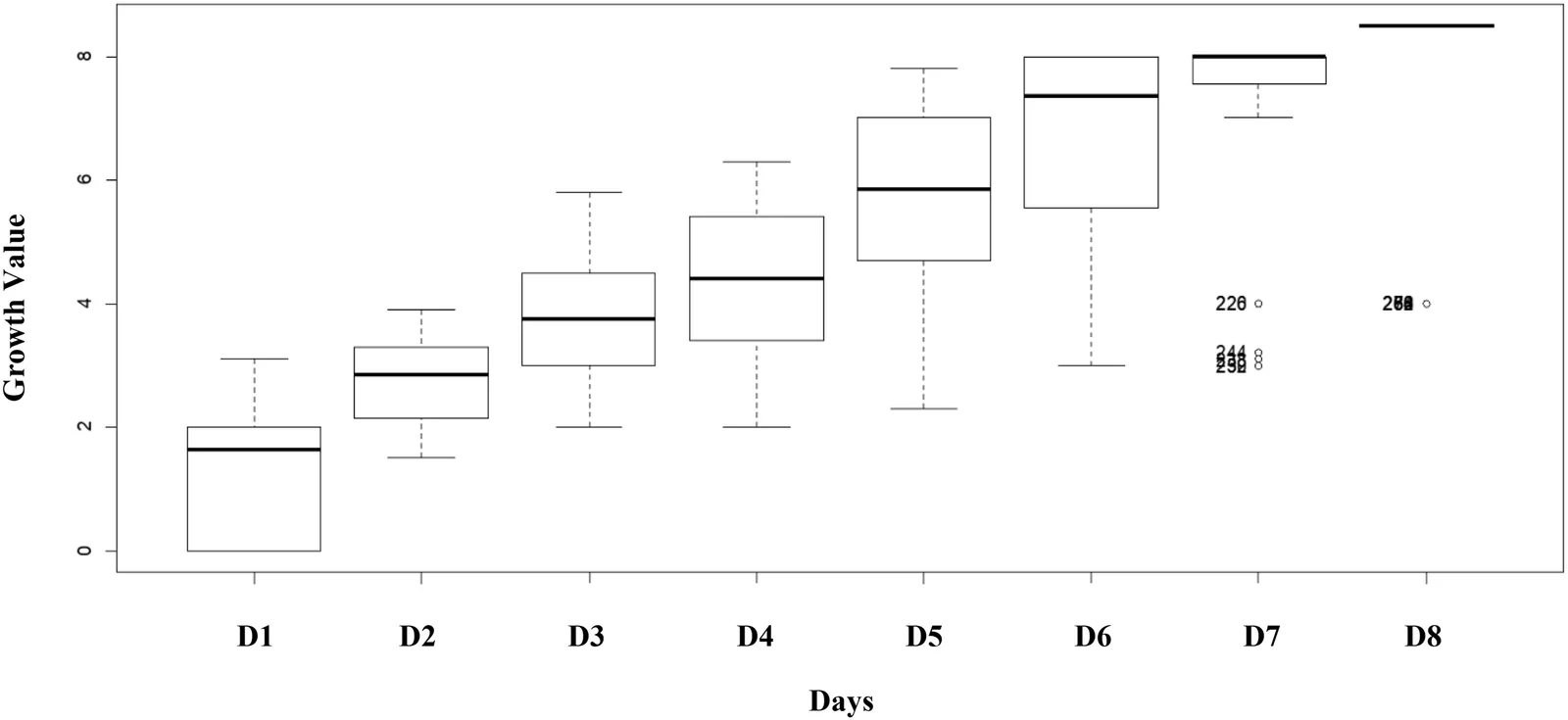
Temporal box plots for Strain N2.

The dispersion and asymmetry seen in (Fig 15) are indicative of both the dynamic of increasing substrate utilization and the temporal and geographical variability of mycelial growth.

**Fig 15:**
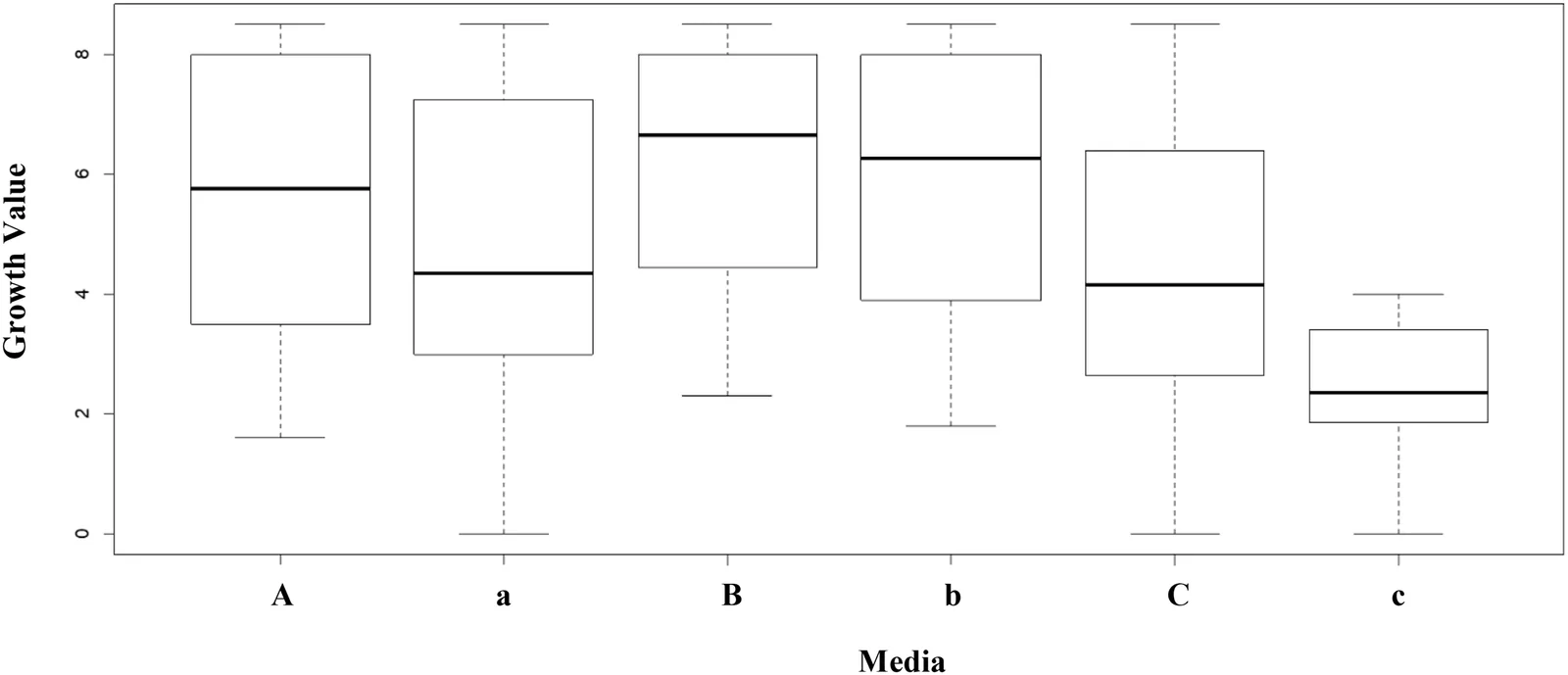
Box plots comparing different culture media for Strain N2. (A): *Algae* with glucose; (a): Algae without glucose; (B): Olive pomace with glucose; (b): Olive pomace without glucose; (C): Potato peels with glucose; (c): Potato peels without glucose.

All three types of media exhibit a notable in mycelial diameter in the graph of averages (Fig 16) indicating that they are suitable for the growth of strain N2. However, after eight days of incubation, the potato peel-based mediums lack of glucose caused a noticeable delay in development as seen by a much smaller growth diameter.

**Fig 16:**
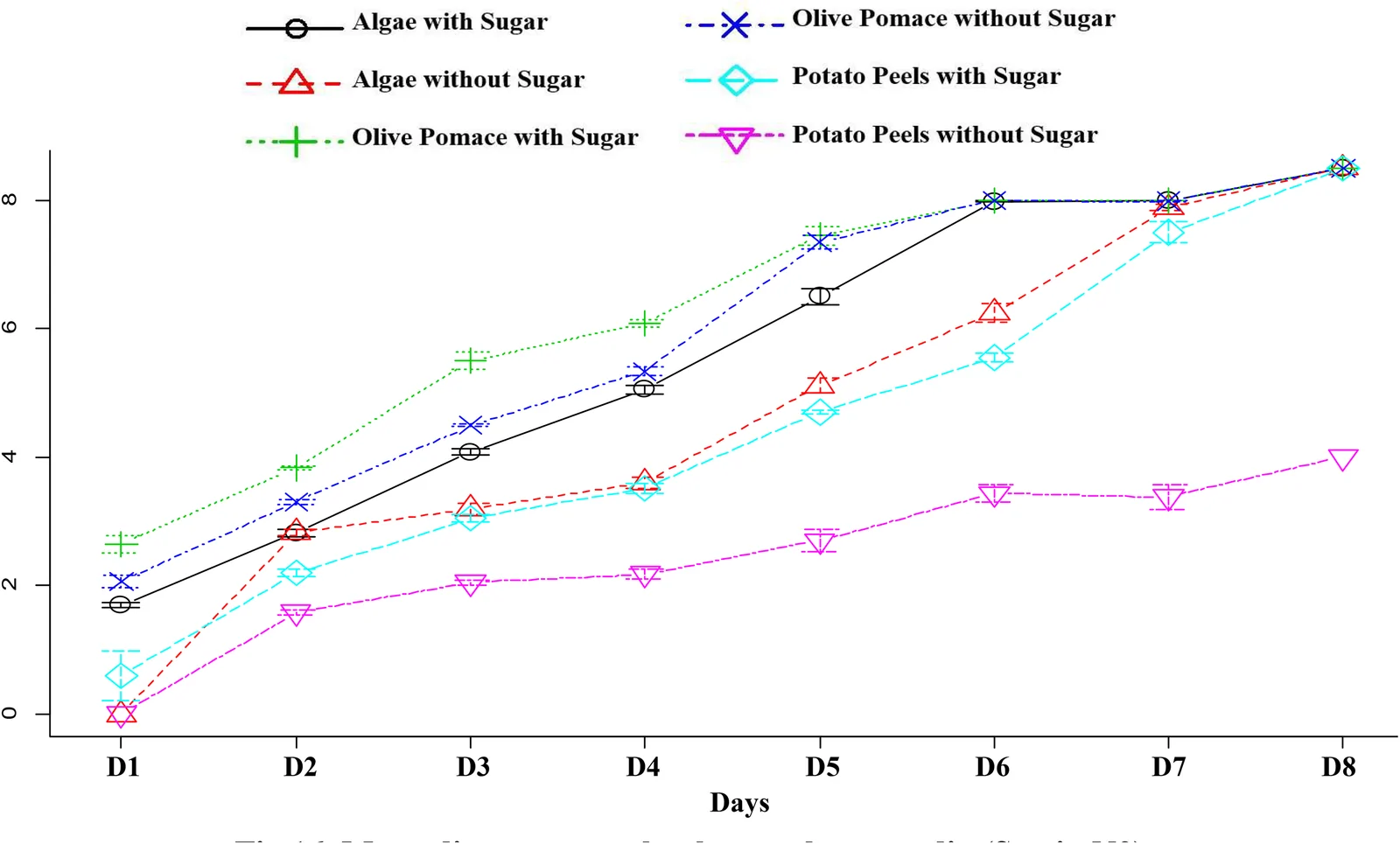
Mean diameters on the three-culture media (Strain N2).

These findings demonstrate once more assimilable carbohydrates influence the rate and severity of fungal growth especially in substrates with low natural sugar content.

Both the time and type of medium factors showed extremely significant differences (P < 2.2x 10^-16^) and a significant interaction between these two parameters. according to multifactorial analysis of variance (ANOVA) (Table 3) these findings illustrate the significance of taking into account both substrate composition and incubation time in an integrated manner when assessing the physiological performance of fungal strains. They also show the combined influence of these two parameters on mycelial growth kinetics.

**Table 3:** Analysis of variance (Strain N2).

Strain N2					
	SumSq	Df	F value	e Pr(>F)	
Diameter	0	1	0,0046	0,9461	
Days	1314.47	7	2749.426	<2^e-16^	***
Medium	463.86	5	1358.316	<2^e-16^	***
Diameter: Days	0.04	7	0.0836	0,999	
Diameter: Medium	0.03	5	0.079	0,9954	
Days: Medium	105.91	35	44.304	<2^e-16^	***
Diameter: Days: Medium	0.44	35	0.1831	1	
Residuals	13.11	192			

Signif. codes: 0 ‘***’ 0.001 ‘**’ 0.01 ‘*’ 0.05 ‘.’ 0.1’ ‘1.

The global outcome shows that our 3-culture medium are ideal for growing filamentous fungus. Especially when glucose is present, they completely meet the nutritional needs required for their growth and development, this finding is particularly significant when compared to widely used commercial media, such as Sabouraud and PDA (potato dextrose agar). In fact, our cultural media seem to provide notable benefits in terms of performance and quality (Fig 17).

**Fig 17:**
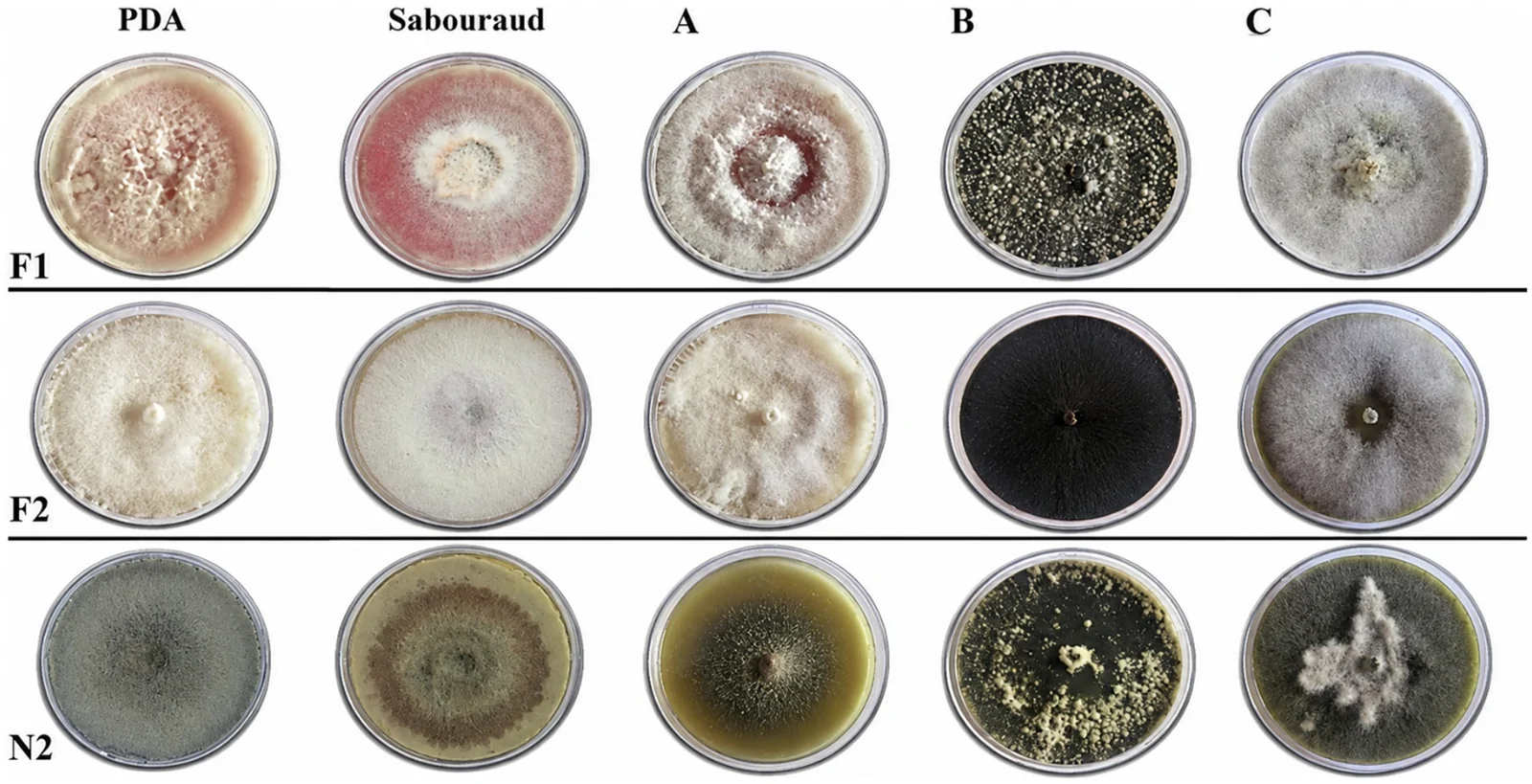
Growth of strains on the three-culture media in comparison with Sabouraud and PDA media. (A): Potato peels medium; (B): Olive pomace medium; (C): Algae medium.

Mycelial growth diameter (cm) on different culture media with and without glucose, compared to PDA and SBA (mean ± SD) are represented in Table 4.

**Table 4:** Mycelial growth diameter (mean ± SD).

Media	N total	Mean	SD	Sum	Min growth (cm)	Median	Max growth (cm)
Olive pomace with glucose	144	6,275	1,86028	903,6	2	6,75	8
Olive pomace without glucose	144	6,0243	2,07227	867,5	1,8	6,65	8
Potato peel with glucose	144	5,6111	2,21483	808	0	6,2	8
Potato peel without glucose	144	1,2194	1,79164	175,6	0	0	6,9
Algae with glucose	144	5,134	2,45038	739,3	0	5	8
Algae without glucose	144	4,3486	2,76974	626,2	0	4,15	8
PDA	144	6,6493	2,3656	957,5	2	7,5	25
Sabouraud	144	6,1326	1,95147	883,1	2	6,8	8

The Commercial reference media PDA and Sabouraud (SAB) represent the highest percentages in the graph, at 19.9% to 19.3% respectively, according to the relative distribution of mycelial growth performance seen across all tested media (Fig 18). this indicates they acknowledged efficacy in the cultivation of filamentous fungi. it is noteworthy. nonetheless that certain experimental media made from Agri-food waste performance that is on par with or even near that of regular media. Therefore, the glucose enriched Algae based medium accounts for 11%, while the glucose enriched olive pomace-based medium and the glucose enriched potato peel based medium each account for 13.2%. These indicate the xylem effect of adding sugar which is a readily metabolized carbon source to enhance the nutrient level of the organic substrate in which the mycelium develops. Without a carbohydrate enrichment, the agro-industrial substrates turned out to be significantly less effective; in fact, the growth yields were lower in olive pomace (12.9%), potato peel (9.3%) and especially in the algae medium (1.2%). This decreased activity may indicate auxotrophy with respect to assimilable carbon sources that may play a crucial role in the regulation of secondary biosynthetic gene clusters and in rapid mycelial metabolism. Notably, however, these basal growth rates compared to the ones obtained from the synthetic controls were found to be very satisfactory. Such a conclusion implies that the combination of remaining fibres and micronutrients found in these food processing residues is enough to support the normal physiological processes. Therefore, the inherent nutrient composition of these wastes is considered to create an optimum microenvironment for fungal growth, even with a slight degree of less effectiveness, and thus it is considered to be a viable alternative to conventional mycological media with both economic and environmental advantages.

**Fig 18:**
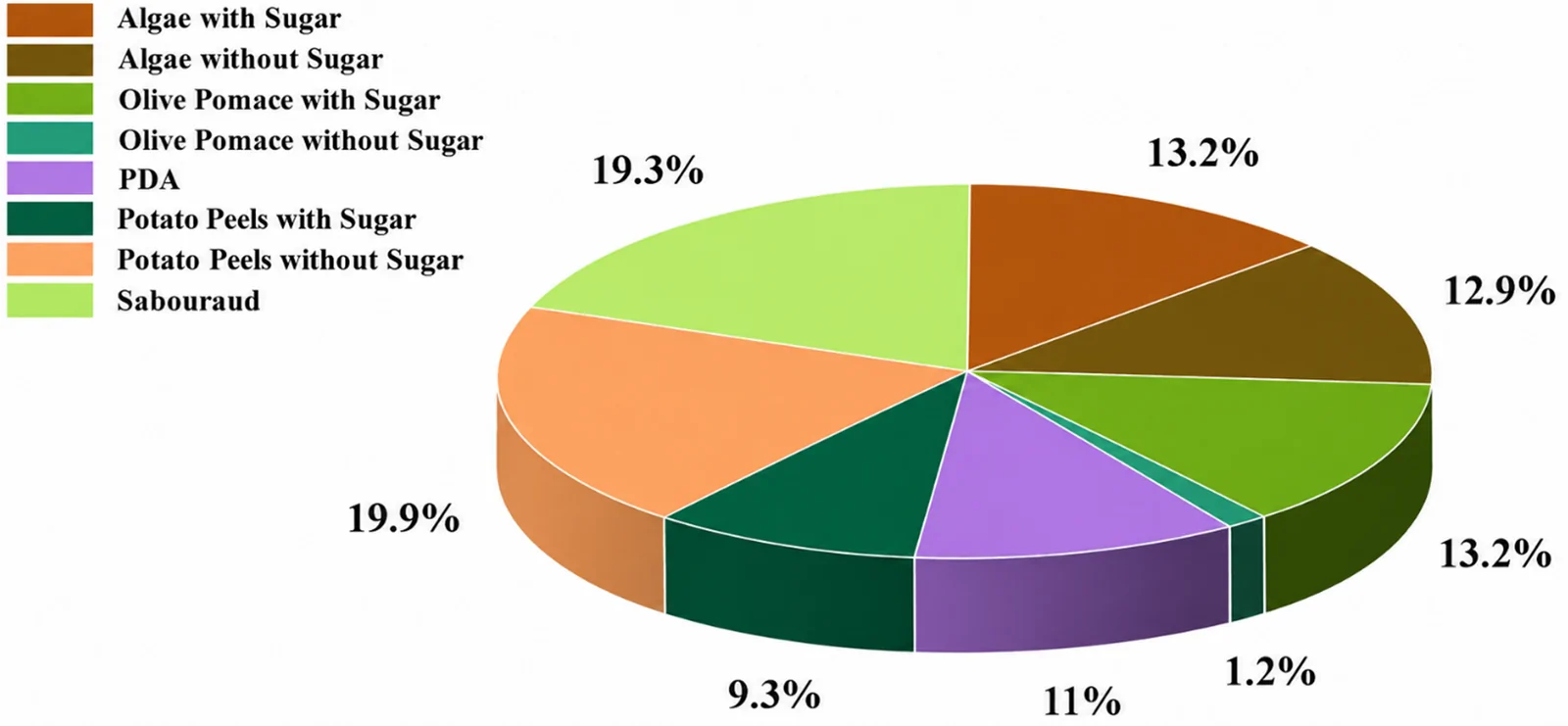
Comparative performance of waste-derived and standard media for filamentous fungi.

The box plot distributions (Fig 19) showed a significant concordance for all the experimental media with or without sugar supplementation with the Sabouraud and PDA media used as the reference media. The overlap of these data shows that the alternative substrates are suitable for the growth of mycelium under conditions similar to those of the standard mycological media, with respect to data spread and symmetry. The similarity in value distribution is an indicator of the similar experimental performance of these waste-based media compared to commercial media.

**Fig 19:**
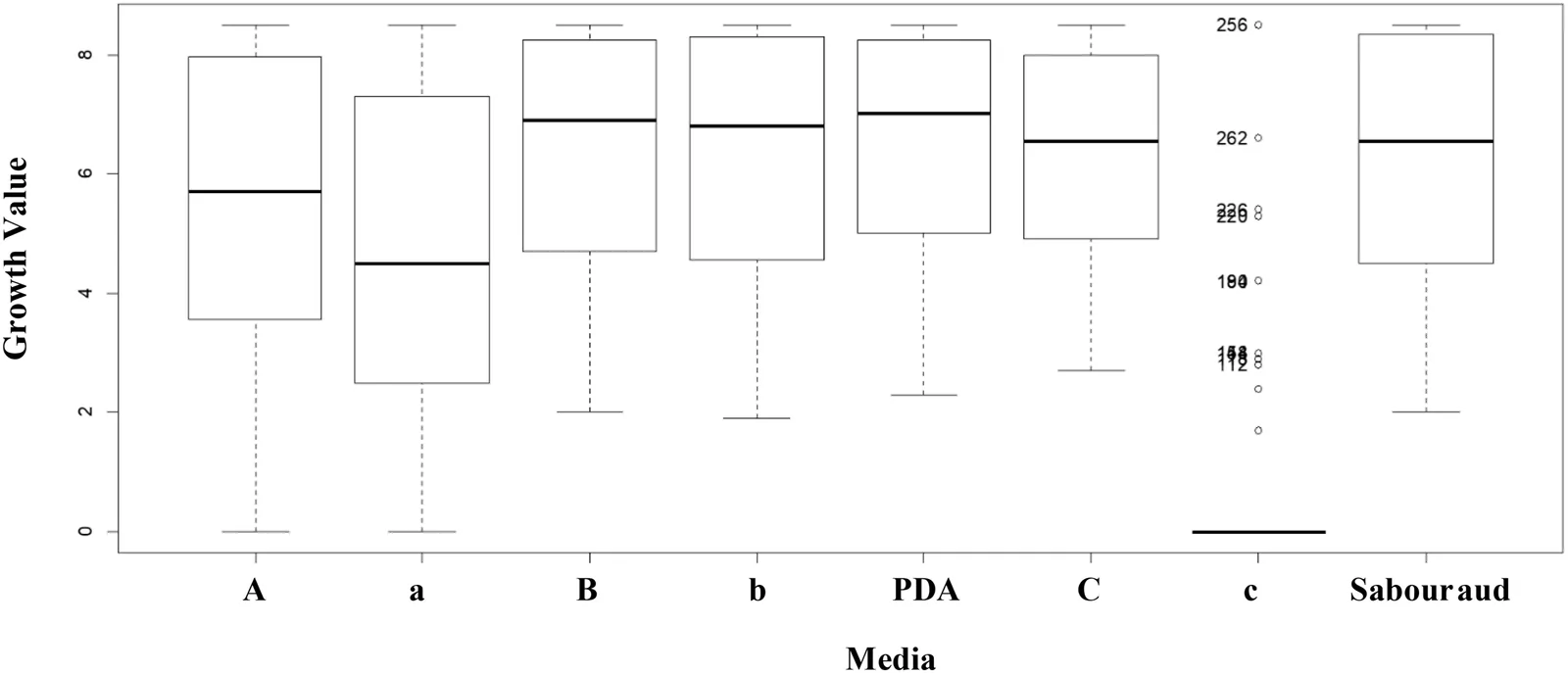
Box plots representing the growth of three strains on various culture media. (A): Algae with glucose; (a): Algae without glucose; (B): Olive pomace with glucose; (b): Olive pomace without glucose; (C): Potato peels with glucose; (c): Potato peels without glucose.

Multifactorial analysis of variance (ANOVA) showed that incubation time had a highly significant effect (P < 2.2 × 10^−16^) on the performance of the various culture media of the three strains. In addition, there was a significant three-way interaction among medium composition, temporal progression, and strain identity (Table 5), indicating that the relationship of the mycelial development kinetics to each of these experimental variables is complex and complex interactions occur. Taken together, the results suggest that the response of fungal species is not only controlled by the chemistry of the substrate, but also by the physiological and temporal synergies that are unique to each species.

**Table 5:** Analysis of variance (Three strains).

All strains					
	Sum Sq	Df	F value	e Pr(>F)	
Diameter	3323.5	71	645.2194	<2.2^e-16^	***
Days	2410.2	61	391.9508	<2.2^e-16^	***
Medium	38.2	3	44.1796	<2.2^e-16^	***
Diameter: Days	382.6	42	31.5647	<2.2^e-16^	***
Diameter: Medium	6.8	21	1.1295	0.3107	
Days: Medium	18.5	7	9.1651	7.02^e-11^	***
Diameter: Days: Medium	39.4	49	2.7872	3.95^e-09^	***
Residuals	207.57	19			

Signif. codes: 0 ‘***’ 0.001 ‘**’ 0.01 ‘*’ 0.05 ‘.’ 0.1’ ‘1.

## Discussion

The present results confirm the high potential for using potato peel, olive pomace and the macroalga *U. lactuca* as alternative substrates for fungal cultivation [32,33]. Their physiological compatibility and nutritional richness with the metabolic demands of *F. oxysporum*, *F. solani* and *A. alternata* were successfully illustrated by their ability to support successful mycelial growth. The observations are in line with the many reports that support the valorization of agro-industrial and environmental biomass for development of sustainable culture media [34–36]. Indeed, multiple investigations have already established that both algal biomass and agro-industrial residues constitute viable sources of carbon and nitrogen, capable of supporting fungal growth and metabolism. Olive pomace, in particular has been found to be a suitable substrate for the synthesis of lignocellulolytic enzymes because of its high polysaccharide and phenolic component content [37]. Similarly, *U. lactuca* is known for its high content of fermentable sugars such as glucose, rhamnose and xylose, which promote microbial growth [35]. The improved growth observed in sugar-supplemented media thus reflects a synergy between the intrinsic nutritional value of organic substrates and the exogenous supply of easily metabolizable carbon. Indeed, the addition of easily assimilable carbon sources, such as glucose or simple sugars, stimulates the growth of filamentous fungi by activating metabolic regulatory mechanisms, in particular carbon catabolic repression (CCR). This mechanism allows fungi to prioritize the use of efficient carbon sources, thereby optimizing their growth and development [38].

### Efficacy of alternative media

The three substrates tested allowed a significant mycelial development, attesting to their richness in macronutrients and essential organic compounds. Olive pomace and *U. lactuca* are characterized by a rich array of polysaccharides, proteins, minerals, fatty acids including oleanolic acid, pentadecanoic acid, cis-vaccenic acid (11E), and stearic acid as well as flavonoids such as isovitexin, astilbin, quercetin, and β-sitosterol, all of which are known to exhibit notable biological activities [39–41]. In contrast, potato peels represent an abundant source of starch and fermentable fibers [42,43]. The balanced carbon:nitrogen ratio which is a natural property of these substrates facilitates fungal growth and production of extracellular enzymes [44]. More recently, it has been found that fungal growth can be promoted by organic waste streams as well. Growth on all substrates was improved by adding glucose, which is a metabolic fuel that can be readily assimilated. This stimulation corroborates the Martin et al. [35] principle of co metabolism, suggesting an increase in energy availability and the kinetic of the mycelium growth by a combination of simple and complex sugars [45].

### Effectiveness of alternative media

The metabolic variety and unique adaptation strategies of filamentous fungus are reflected in the variations between the three fungal species [46] *F. oxysporum* demonstrated the ability to take use of complicated lignocellulosic substrate by growing vigorously on all media, including those without glucose. *F. solani*, in contrast, was more limited in its growth capacity which depended on readily available carbohydrates, suggesting a smaller initial (more limited) enzymatic ability to break down complex polymers. While its growth was hinder on sugar poor media. The most tolerant species was *A. alternata*. The observations are consistent with the direct relationship of substrate chemical composition and carbon bioavailability with growth dynamics [47].

Our study findings are in line with recent studies on the adaptability of fungi to agricultural waste substrates. To optimize the solid-state fermentation substrates for *Trichoderma asperellum* [48], used an experimental design with the admixtures that contained agricultural wastes, such as olive pomace, potato flakes and vine shoots, which had a significant effect on the biosynthesis of the antifungal compound 6-pentyl-alpha-pyrone. Their work demonstrated that the ideal substrate formulation for each strain was different, and we found that *F. oxysporum* grew better on the lignocellulosic substrates than did *F. solani*, supporting our observation. Moreover, they emphasize the enzymatic adaptability of the fungi used in this study, especially their ability to adjust the production of lignocellulolytic enzymes in order to adapt to the nutritional conditions [37,49].

### Performance of alternative media compared to commercial

The organic waste-based media gave better growth performance than the conventional commercial media PDA and Sabouraud when glucose was added to the media. The formulations made from olive pomace and potato peel had radial growth rates similar to the PDA and Sabouraud mediums attesting to their ability to reproduce nutritional and physicochemical characteristics of classic mycological media [34,50]. In addition, all of these alternatives were characterized by being homogenous, thermally stable and possessing suitable gelling properties after the sterlization process, which would allow for utilization in a wide variety of experimental setups. These attributes allow them to be used as an economical and environmentally friendly alternative to conventional laboratory tasks.

Angel and Ravimannan [51] evaluated vegetable waste including cabbage, onion, carrot, and beans as alternative nutrient sources for six fungal genera: *Aspergillus* sp., *Fusarium* sp,. *Mucor* sp., *Penicillium* sp., *Rhizopus* sp. and *Trichoderma* sp, finding that several waste-based media exhibited superior performance compared to Potato Dextrose Broth. More recently, Ajiboye et al. [26] demonstrated that yam and potato peel-based media effectively supported the growth of six fungal species, with colony diameters comparable to control PDA. Proximate analysis of the substrate revealed high carbohydrate content, moderate protein and abundant minerals, confirming potato peels as a cost-effective, nutrient-rich substrate for fungal cultivation.

It’s crucial to draw attention to a few restrictions on the application of the substitute medium in fungal identification processes. Nevertheless, the macroscopic appearance of the mycelium can be changed by the substrate texture, color, and organic composition especially with regard to pigmentation, density, and relief. These morphological differences can be confusing to mycologists during macroscopic identification, where the appearance of colonies is a crucial diagnostic criterion, even if they have no effect on viability or growth [52,53]. For Post identification procedures like subculturing, strain preservation, biomass generation or enzyme testing. It is advised to set aside alternate media to guarantee morphological dependability and comparability of taxonomic observations. traditional media like PDA and Sabouraud should be used for the early phases of identification. an ideal experimental framework is provided by the complementarity between standard and alternative media: standard media provide diagnostic accuracy while alternative media allow for cost-effective and sustainable cultivation without sacrificing biological performance. This differentiation in application encourages the practical incorporation of these novel media into mycological procedures fusing sustainability with scientific rigor.

### Ecological and Biotechnological

These substrates are used in fungal Biotechnology,which directly supports the adoption of a circular economy and lessens the environmental impact of marine trash and agree food [54,55] in addition to limiting ecosystem degradation, their recovery lowers production costs related to the acquisition of commercial media [56] these findings support the usefulness of partial substitution techniques meant to include renewable and local resources into microbiological processes [57,58].

Recent studies have additionally proven the biotechnological use of waste valorization. Sar et al. (2025) [19] studied the production of Aspergillus oryzae in the presence of agro-industrial derived volatile fatty acids (VFAs) and found that dry biomass production was 0.41 g/g VFAs fed, similar to the crude protein content of conventional protein sources in continuous-feeding fed-batch mode. This is an indication of the potential of substrates in sustainable production of animal feed ingredients from waste.

Likewise, Zuffi et al. [59] investigated the utilization of grape pomace, a by-product with similar chemical composition to olive pomace, for Pleurotus mushroom cultivation, focusing on its phenolic and fiber content. Elemental analysis has shown high C contents (46%), N contents (2.5%) and a high content of macro- and microelements (P, Ca, Mg, Fe) than conventional substrate. The fresh weight of mushrooms increased statistically significantly after adding grape pomace, by over 130% in some instances. This indicates similar results with ours that allows a broader applicability of the valorization of agro-industrial waste for sustainable fungal cultivation. This novel approach will reduce reliance on costly synthetic media, contribute to sustainability and open doors to biotechnology and applied microbiology.

## Conclusion

The present study proves that the conventional mycological media can be replaced by formulations prepared from organic wastes, namely potato peel residues, olive pomace and biomass of *U. lactuca* organic macroalga, for filamentous fungi cultivation, from scientific and practical point of view. These substrates were shown to have exceptional potential for biotechnological application and support fungal growth at levels identical to those observed on PDA and Sabouraud agar.

In addition to their biological activity, the tested formulations proved to have good physical stability and handling properties, thus they can be used as alternative formulations that are environmentally friendly and economically viable for large-scale bioprocesses. There was however a practical limitation to be taken into account: deviations in texture, pigmentation and overall chemical composition from normal media were found to have a subtle effect on colonies of mycelium at the macro level. The constraint is not as significant for taxonomic applications based on morphological criteria (although not as important as the second use of the substrates, which are still useful for applications such as strain maintenance, preservation, preparation of inocula, functional screening, and biomass production). Conceptually this paper serves as a real-world example of the principles of circular bioeconomy, where waste and unused organic matter is turned into actionable inputs for microbiological research and industry. At a deeper level, it calls for a shift in thinking about how culture media are used, from being strictly commercial formulations to more flexible and resource-hungry approaches that take advantage of local resources and environmental sustainability. Future studies should include extensive physicochemical characterization, evaluation of these substrates under both submerged and solid-state fermentation conditions, and the application of these substrates to a large panel of fungal strains from a variety of phylogenetic groups. These investigations will play an important role in supporting the validity of these media and make it easier to translate media from laboratory scale experiments to industrial platforms.

## Acknowledgments

The authors would like to acknowledge the Deanship of Graduate Studies and Scientific Research, Taif University for funding this work.
